# Disability and Older Age Return Migration: Evidence Against the Salmon Bias

**DOI:** 10.1093/geroni/igab046.1754

**Published:** 2021-12-17

**Authors:** Mara Sheftel

**Affiliations:** Penn State University, Brooklyn, New York, United States

## Abstract

Mexican immigrants make up an increasing proportion of the US population 65 and older. Whereas this population has among the lowest rates of disability at working ages, there is growing evidence of high rates of disability at older ages, findings which contradict what mechanisms of selection, namely the “salmon bias,” would predict. However, largely due to data limitations disability rates between those who stay in the US into older ages and those who return to Mexico are rarely compared. Here two waves of data from the US based Health and Retirement Study and the Mexican Health and Aging Study are combined to create a novel dataset that enables an interrogation of the widely held assumption of negative selection on health among return migrants. Investigating three measures of functional limitation and disability, results show higher prevalence of disability for stayers as compared to both younger and older returnees. These results are robust to controls for childhood background, adult socioeconomic status, and migration related variables and hold for those who immigrated during different immigration policy regimes. These findings are novel not only because they stand in opposition to previous assumptions about the direction of health selective return migration, but also because they mean that those remaining in the United States into older ages are among the most vulnerable.

